# AFC1 Compound Attenuated MI/R-Induced Ventricular Remodeling *via* Inhibiting PDGFR and STAT Pathway

**DOI:** 10.3389/fphar.2019.01142

**Published:** 2019-10-15

**Authors:** Jie Liu, Xiaohui Zhou, Qingshu Meng, Kevin W. Huang, Jing Liu, Jinjun Tie, Rulin Zhuang, Guohan Chen, Yuhui Zhang, Lu Wei, Li Huang, Chun Guang Li, Binghui Wang, Huimin Fan, Zhongmin Liu

**Affiliations:** ^1^Research Center for Translational Medicine, Shanghai East Hospital, Tongji University School of Medicine, Shanghai, China; ^2^Department of Cardiovascular and Thoracic Surgery, Shanghai East Hospital, Tongji University School of Medicine, Shanghai, China; ^3^Shanghai Heart Failure Research Center, Shanghai East Hospital, Shanghai, China; ^4^Monash Centre of Cardiovascular Research and Education in Therapeutics, Department of Epidemiology and Preventive Medicine, Monash University, Melbourne, VIC, Australia; ^5^Department of Ultrasound, Shanghai East Hospital, Tongji University School of Medicine, Shanghai, China; ^6^NICM Health Research Institute, Western Sydney University, Westmead, NSW, Australia; ^7^Department of Heart Failure, Shanghai East Hospital, Tongji University School of Medicine, Shanghai, China

**Keywords:** AFC1 compound, myocardial ischemia reperfusion, platelet derived growth factor, platelet derived growth factor receptor, ventricular remodeling

## Abstract

**Background:** Effective interventions to improve the outcome of patients subjected to myocardial ischemia reperfusion (MI/R) are urgent in clinical settings. Tanshinone IIA (TSA) is reported to attenuate myocardial injury and improve ventricular remodeling post MI/R. Here, we evaluated the efficacy of AFC1 compound that is similar to TSA structure in murine MI/R models. We found that AFC1 had a comparable effect of improving murine cardiac function after MI/R while it was superior to TSA in safety profile. Administration of AFC1 reduced reactive oxygen species (ROS) production, inflammatory cells infiltration, and the expression of platelet derived growth factor receptors (PDGFR) in infarcted myocardium. Treatment with AFC1 also attenuated MI/R-induced cardiac remodeling and contributed to the recovery of cardiac function. Additionally, AFC1 reversed the elevation of PDGFR expression induced by PDGF-AB in both neonatal rat cardiomyocytes (NCMs) and neonatal rat cardiac fibroblasts (NCFs) and suppressed PDGF-AB induced NCM hypertrophy *via* STAT3 pathway and NCF collagen synthesis through p38-MAPK signaling *in vitro*. Similarly, AFC1 may contribute to the recovery of cardiac function in mice post MI/R *via* suppressing STAT signaling. Our results confirmed that AFC1 exerts anti-hypertrophic and anti-fibrotic effects against MI/R-induced cardiac remodeling, and suggest that AFC1 may have a promising potential in improving the outcome of patients who suffered from MI/R.

## Introduction

Ischemic heart disease (IHD) has been a leading cause to high morbidity and mortality in developed countries with increasing incidence in developing countries (Chan et al., 2011). Although timely intervention can restore coronary flow, the reperfusion process triggers myocardium injury (Yellon and Hausenloy 2007), known as myocardial ischemia reperfusion (MI/R) injury, and cardiac remodeling, and subsequent heart failure (HF), which are the predominant contributors of death worldwide (Murdoch et al., 2006; Sun, 2009; Wang et al., 2015a). Therefore, effective prevention and treatment strategies to attenuate or reverse MI/R-induced remodeling is of great clinical value in IHD patients.

Recently, the protective role and relatively less adverse reaction of traditional Chinese medicine (TCM) in IHD have been highlighted. *Danshen,* the dry roots and rhizome of *Salvia miltiorrhiza Bge.*, has been widely used either alone or in combination with other herbal ingredients for patients with IHD and other cardiovascular diseases in both China and other countries because of its efficacy in improving microcirculation and protecting against myocardial ischemia (Cheng, 2007). For example, results from meta-analysis demonstrated the potential benefits of compound *Danshen* dripping pill (CDDP) for treating coronary heart disease (Luo et al., 2015; Huang et al., 2016). However, the overall quality of the evidences in the systematic reviews was poor and high-quality evidence is warranted to support the clinical application of CDDP in treating IHD. Tanshinone IIA (TSA) is the most abundant and active diterpenoid quinone compound among lipophilic components extracted from *Danshen* (Wu et al., 2013). It is reported that TSA can attenuate myocardial injury and improve ventricular remodeling post MI/R *via* reducing reactive oxygen species (ROS) generation in mitochondria (Zhou et al., 2003; Jin and Li, 2013; Jin et al., 2013). However, in clinical settings, the efficacy of TSA is limited because of its lipid-soluble property, low bioavailability, and short half-life (Liu et al., 2013). Therefore, TSA modification targeting the above shortcomings is a promising strategy for its development in MI/R therapy.

Recently our team has investigated various compounds with similar core structure of TSA, including AFC1. In this study, we proved its potential role in cardiac protective effect against cardiac cell injury, hypertrophy, and fibrosis *in vitro* and *in vivo*. Thus, AFC1 compound may become a novel therapeutic pharmaceutical for patients subjected to MI/R. Accordingly it is important to study the effect of AFC1 *in vivo* to evaluate its efficacy and possible mechanism of actions.

Previous studies have demonstrated the importance of growth factors in IHD (Liu et al., 2014; Pello et al., 2015). High level of platelet derived growth factor (PDGF) in infarcted hearts contributed to myocardial inflammation and fibrosis in rats (Zhao et al., 2011). PDGF family is composed of four kinds of isoforms, -A, -B, -C, and -D, which comprised homodimers of PDGF-AA, -BB, -CC, and –DD and heterodimer of PDGF-AB (Price et al., 2003). PDGF exerts its biological activities through two distinct subtypes of tyrosine kinase receptors, PDGF receptors (PDGFR)-α and -β expressed on cardiomyocytes (Vantler et al., 2010). Excessive expression of PDGF could result in deposition of extracellular matrix and further induces cardiac remodeling (Vantler et al., 2010; Zhao et al., 2011). PDGF could induce H_2_O_2_ (kind of ROS) generation in mouse embryonic fibroblasts (MEFs) by binding PDGFR (Choi et al., 2005). On the other hand, the inhibition of PDGF/PDGFR pathway could attenuate the vascular remodeling *via* reducing the inflammatory response in the hypertensive rat with myocardial fibrosis (Fan et al., 2013). Therefore, PDGF/PDGFR may promote the development of cardiac remodeling after MI/R by mediating oxidative stress and inflammatory response.

Our present study demonstrated for the first time that treatment with AFC1 compound effectively attenuates MI/R-induced cardiac remodeling, accompanied by decreased PDGFR expression, oxidative stress, and inflammatory response in hearts post MI/R. Moreover, AFC1 compound inhibited NCM hypertrophy and NCF collagen synthesis induced by PDGF-AB and contributed to the recovery of cardiac function post MI/R *via* regulating STAT3 pathway.

## Materials and Methods

### Animals

Specific pathogen-free, male C57BL/6 mice (8–10 weeks) were purchased from Slac Laboratory Animal Co. Ltd (Shanghai, China). All experiments were conducted in accordance with protocols approved by the Institutional Animal Care and Use Committee of Tongji University.

### Establishment of Myocardial Ischemia Reperfusion Murine Models

Echocardiography was performed before the establishment of MI/R models. Mice with EF above 50% were included in the *in vivo* experiment. MI/R models were established as described previously (Pu et al., 2013). Regional ischemia was achieved by ligation of LAD using a 10-0 silk suture with a section of silica gel tube. Successful myocardial ischemia was achieved when the anterior wall of the left ventricular (LV) turned pale. After 30 min of ischemia, the ligation was relieved and the successful reperfusion was confirmed by epicardial hyperemia. TSA (5 mg/kg) or AFC1 compound (7 or 14 mg/kg) were intraperitoneally administrated daily for 1 week following MI/R. Mice with the heart exposed through left thoracic incision without ligation of left anterior descending coronary artery (LAD) were included in the sham group. Mice with LAD ligation for 30 min and then reperfusion for 2 weeks were randomly assigned to the following groups (5 mice in each group): Sham, MI/R, MI/R+TSA (5 mg/kg), MI/R+AFC1-L (7 mg/kg), and MI/R+AFC1-H (14 mg/kg). Each experiment was repeated at least three times. The AFC1 compound was synthesized by CG LI’s lab at Western Sydney University.

### Echocardiography

On day 14 post MI/R, the mice were anesthetized using isoﬂurane then M-mode echocardiography was performed in mice with echocardiographic imaging system (Visualsonics, Canada) equipped with a 15-MHz linear transducer. Parameters of cardiac function were measured digitally on the M-mode tracings. All echocardiographic procedures were performed by a qualified investigator who was blinded to the grouping and treatment. The long-axis and short-axis view in B-mode were obtained. The B-mode guided M-mode view at the papillary muscle level was obtained for the evaluation of parameters. The end-systolic and end-diastolic LV dimensions were captured to calculate the LV ejection fraction (EF) and fractional shortening (FS) as previously described (Lin et al., 2013).

### Histology

After the reperfusion, fresh heart biopsies were fixed in 4% paraformaldehyde overnight at 4°C and embedded in paraffin. Sections into 5-μm slices were stained with hematoxylin-eosin (H&E) or Masson’s trichrome for assessment of fibrosis. Tissues for immunofluorescence were submerged in liquid nitrogen and then embedded in optimal cutting temperature (OCT) solution (Sakura Finetek, USA) on dry ice to be frozen completely. Cardiomyocyte hypertrophy was examined in the peri-infarct zone. Myocyte cross-sectional areas were measured using Image J software (National Institutes of Health) in frozen sections stained with 5 μg/ml wheat germ agglutinin (WGA-Alexa Fluor^®^ 488 conjugate, Invitrogen, USA). Five parts were chosen in the WGA images (200X) including left top, right top, middle, left bottom, and right bottom, and six cells were analyzed for each part. In other experiments, the hearts were excised for Masson staining to evaluate the cardiac remodeling. For inflammatory cell infiltration and PDGFR protein expression, immunofluorescence staining with anti-CD45 (Cell Signaling Technology, USA) and anti-PDGFRα (Cell Signaling Technology, USA) in frozen sections was conducted. Then the number of CD45+ cells/field were quantified by Image J software (National Institutes of Health, USA).

### ROS Production

ROS production was evaluated with dihydroethidium (DHE, Sigma, USA) on frozen myocardial sections. Heart slices were incubated at 37°C for 30 min with 10 μmol/L DHE in phosphate-buffered saline (PBS). Staining was captured by fluorescence microscope (Leica, Germany). Fluorescence intensity was quantified by using Image J software (National Institutes of Health, USA).

### Isolation and Culture of Primary Cardiomyocytes and Cardiac Fibroblasts

NCM and NCF were isolated from 1-day-old pups with enzymatic digestion as described previously (Thomas et al., 2002; Tzanidis et al., 2003). Purified NCMs were seeded at high density of 1×10^6^ in 6-well plate and 3×10^5^ in 12-well plate and maintained in serum-free DMEM (Gibco, USA) supplemented with 5 mg/ml insulin, 10 mg/ml apo-transferrin, and 50 mM KCl. Bromodeoxyuridine (0.1 mM, Sigma, USA) was applied for the first 3 days. NCF were seeded at a density of 3×10^5^ in 6-well plate and 5×10^4^ in12-well plate and cultured in DMEM HG supplemented with 0.5% BSA and 1% L-ascorbic acid (Sigma, USA). On the fourth day, 1 h after pretreatment with AFC1 (0.1, 1.0, 3.0, 10.0 μM), PDGF-AB (10 ng/ml, PEPROTECH, USA) was added to induce hypertrophy in NCM and collagen synthesis in NCF. After 48 h of PDGF-AB stimulation, cells were harvested to determine hypertrophy and fibrosis, defined as a significant increase in protein content *via* ^3^H-Leucine or ^3^H-Proline incorporation. Cells were treated with AFC1 or DMSO for 1 h and then PDGF-AB for another 15 min for protein sample collection and Western blot detection.

### ^3^H-Leucine and ^3^H-Proline Incorporation

On the fourth day, after addition of PDGF-AB, NCMs were labeled with ^3^H-Leucine (1 μCi) and NCF with ^3^H-Proline (5 μCi) (PerkinElmer, USA) for 48 h. The experiment was terminated by washing the cells with cold PBS for three times then precipitating with 10% trichloroacetic acid (TCA, Sigma, USA) for 30 min. Cells were then lysed in 1 M NaOH overnight in 4°C. After neutralization with 1 M HCl and addition of scintillation fluid (PerkinElmer, USA), radioactivity was captured in a liquid scintillation counter (HIDEX 300 SL, Finland). The results represent at least three separate experiments done in triplicate for each condition.

### MTT Assay

Cell viability was determined with colorimetric method using the MTT assay. NCFs were seeded at a density of 1.5×10^3^ and human umbilical vein endothelial cells (HUVECs, PromoCell) at 1×10^4^ cells per well in 96-well plate. After treatments for 48 h, cells were incubated with 10 μl of 5 mg/ml MTT (3-(4,5-dimethyl-2-thiazolyl)-2,5-diphenyl-2-H tetrazolium bromide (Sigma, USA) solution at 37°C for 4 h. The formazan crystals were dissolved in 100 μl of isopropanol for 20 min at 37°C, and absorbance at 570 nm was detected on a Microplate Reader (SpectraMax, USA).

### Quantitative RT-PCR (q-PCR)

Total RNA (1 μg) extracted from myocardium, NCM, and NCF were reverse transcribed with PrimeScript RT reagent Kit with gDNA Eraser (TaKaRa, Japan), and q-PCR was performed on the 7900HT Fast Real Time PCR System (Applied Biosystems, UK) with the SYBR mastermix (Applied Biosystems, UK). All primer sequences were listed in **Table 1**.

**Table 1:** Quantitative polymerase chain reaction primers.

Gene	Forward primer (5’-3’)	Reverse primer (5’-3’)
mPDGF-A	GAGGAAGCCGAGATACCCC	TGCTGTGGATCTGACTTCGAG
mPDGF-B	CATCCGCTCCTTTGATGATCTT	GTGCTCGGGTCATGTTCAAGT
mPDGFR-α	ACACGTTTGAGCTGTCAACC	CCCGACCACACAAGAACAGG
mPDGFR-β	TTCCAGGAGTGATACCAGCTT	AGGGGGCGTGATGACTAGG
mIL-1β	CGAGGCTAATAGGCTCATCT	GTTTGGAAGCAGCCCTTCAT
mTNF-α	AGCCGATGGGTTGTACCTTGTCTA	TGAGATAGCAAATCGGCTGACGGT
mIL-6	TGATGCACTTGCAGAAAACA	ACCAGAGGAAATTTTCAATAGGC
mGAPDH	AACTTTGGCATTGTGGAAGG	ACACATTGGGGGTAGGAACA
rPDGF-A	TTCTTGATCTGGCCCCCAT	TTGACGCTGCTGGTGTTACAG
rPDGF-B	GCAAGACGCGTACAGAGGTG	GAAGTTGGCATTGGTGCGA
rPDGF-C	CAGCAAGTTGCAGCTCTCCA	GACAACTCTCTCATGCCGGG
rPDGF-D	ATCGGGACACTTTTGCGACT	GTGCCTGTCACCCGAATGTT
rPDGFR-α	GCTACACGTTTGAGCTGTCAAC	ATGGTGGTCATCCACAAGC
rPDGFR-β	TCTCTCATCATCCTCATCATGC	CCTTCCATCGGATCTCATAGC
rANP	GAGGAGAAGATGCCGGTAG	CTAGAGAGGGAGCTAAGTG
rα-SKA	GCATGCAGAAGGAGATCACA	CATAGCACGATGGTCGATTG
rβMHC	AGATCGAGGACCTGATGGTG	GATGCTCTTCCCAGTTGAGC
rCol Type I	CATGTTCAGCTTTGTGGACCT	GCAGCTGACTTCAGGGATGT
rCol Type III	GGTCACTTTCACTGGTTGACGA	TTGAATATCAAACACGCAAGGC
rGAPDH	ACAAGATGGTGAAGGTCGGTG	AGAAGGCAGCCCTGGTAACC

Comparison of gene expression in different samples was calculated as follows. Each sample was related to an internal control gene (GAPDH). For example, Sample A was the control sample and Sample B was the treated one.

ΔΔCt = (Ct gene of interest - Ct _GAPDH_)sample B - (Ct gene of interest - Ct _GAPDH_)sample A.

Finally, relative quantification of gene expression (Sample B) = 2^-ΔΔCt^.

### Western Blot

Protein was extracted from NCM, NCF, and homogenized myocardium tissue in lysis bu?er. Protein lysate concentrations were determined *via* Pierce BCA Protein Assay Kit (Termo Scientific, USA). Equal amount of protein sample (20–30 μg/lane) from each group was subjected to 10% SDS-PAGE and transferred onto nitrocellulose membranes. After blocking with 5% bovine serum albumin (BSA), membranes were incubated overnight with primary antibodies (1:1,000, Cell Signaling Technology, USA) against p-JAK2, p-STAT3, STAT3, p-p38, GAPDH, and pan-actin. On the second day, the membranes were incubated with fluorescent secondary antibody DyLight 800-Goat Anti-Rabbit IgG (H+L) (KPL, USA). The membranes were scanned by ODYSSEY infrared imaging system (LI-COR Biosciences, USA). After incubation of phosphorylated proteins, we used stripping buffer (beyotime, China) to extract antibodies. Then we did the blocking and the following incubation procedures to obtain total protein quantification.

### Statistical Analysis

Data, all presented as mean ± SEM, were analyzed using SPSS software, version 11.0 (SPSS Inc., Chicago, IL, USA). For *in vivo* experiments, the Mann-Whitney U test was used for comparisons between different groups. One-way analysis of variance with a Bonferroni *post hoc* test was used for multiple comparisons. P < 0.05 was considered statistically significant.

## Results

### Safety Profile of AFC1 *In Vivo*

**Figure 1:**
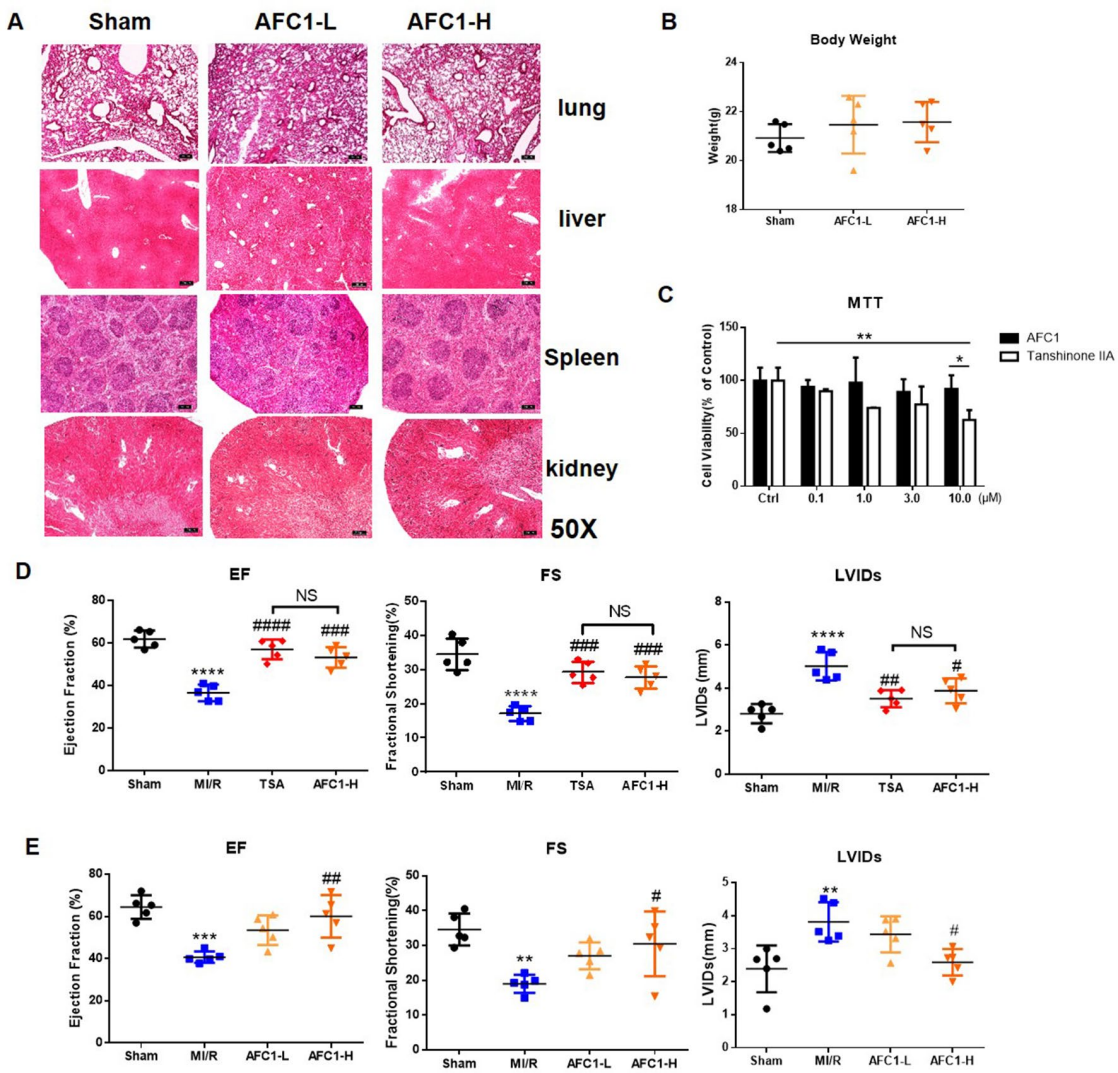
Administration of AFC1 contributed to the recovery of cardiac function after MI/R in murine models. Mice were injected with low (7 mg/kg), high-dosage (14 mg/kg) of AFC1 compound or TSA (5 mg/kg) intraperitoneally for 7 days after MI/R (n = 5). **(A)** Lung, liver, spleen, and kidney sections were harvested on day 14 for H&E staining. Figures showed the representative data. Magnification was ×50. **(B)** Body weight change of mice after AFC1 administration was analyzed on day 14. **(C)** HUVECs were seeded at a concentration of 1×10^4^/well in 96-well plate and treated with DMSO, AFC1 compound, or TSA for 24 h. Then cell viability was evaluated by MTT assay. **(D)** EF, FS, and LVIDs were measured by echocardiography on day 14 following MI/R. **(E)** Average values for EF, FS, and LVIDs. Each experiment was repeated for at least three times and results indicated mean ± SEM of one independent experiment. *p < 0.05, **p < 0.01, ***p < 0.001, ****p < 0.0001 versus sham or selected group; ^#^p < 0.05, ^##^p < 0.01, ^###^p < 0.001 versus MI/R. *represent the significant different between MI/R group vs the sham group; ^#^represent the significant different between treatment group and MI/R group.

To determine the safety of AFC1 *in vivo*, we evaluated the pathology of liver, kidney, spleen, and lung from mice administrated with AFC1 compound for 14 days. As shown by **Figure 1A**, there are no significant morphological changes in these organs. Besides, AFC1 administration did not change the body weight of mice on day 14 (**Figure 1B**). Previous study showed the cytotoxic effect of TSA on human umbilical vein endothelial cells (HUVECs) in a dose-dependent manner (Nizamutdinova et al., 2012). Then we treated the HUVECs with AFC1 or TSA. MTT data showed no difference in HUVECs viability between AFC1 and TSA group when both concentrations are 0.1, 1.0, and 3.0 µM (**Figure 1C**). However, AFC1 treated cells showed higher viability than the TSA group at 10 µM (p < 0.05) (**Figure 1C**).

### Administration of AFC1 Contributed to the Recovery of Cardiac Function After MI/R in Murine Models

Further cardiac function data showed that both high dose AFC1 (14 mg/kg) compound and TSA (5 mg/kg) significantly improved the EF and FS and systolic left ventricular interior diameters (LVIDs) in MI/R hearts (**Figure 1D**). However, there was no significance when AFC1 and TSA treated groups were compared. Then we assessed whether low (7 mg/kg) and high dose (14 mg/kg) of AFC1 have equal protective role in murine MI/R models. As shown by **Figure 1E**, only high dose of AFC1 increased the EF as well as FS and decreased LVIDs compared to the MI/R group on day 14 post operation.

### AFC1 Compound Attenuated Mi/R-Induced Cardiac Remodeling

**Figure 2:**
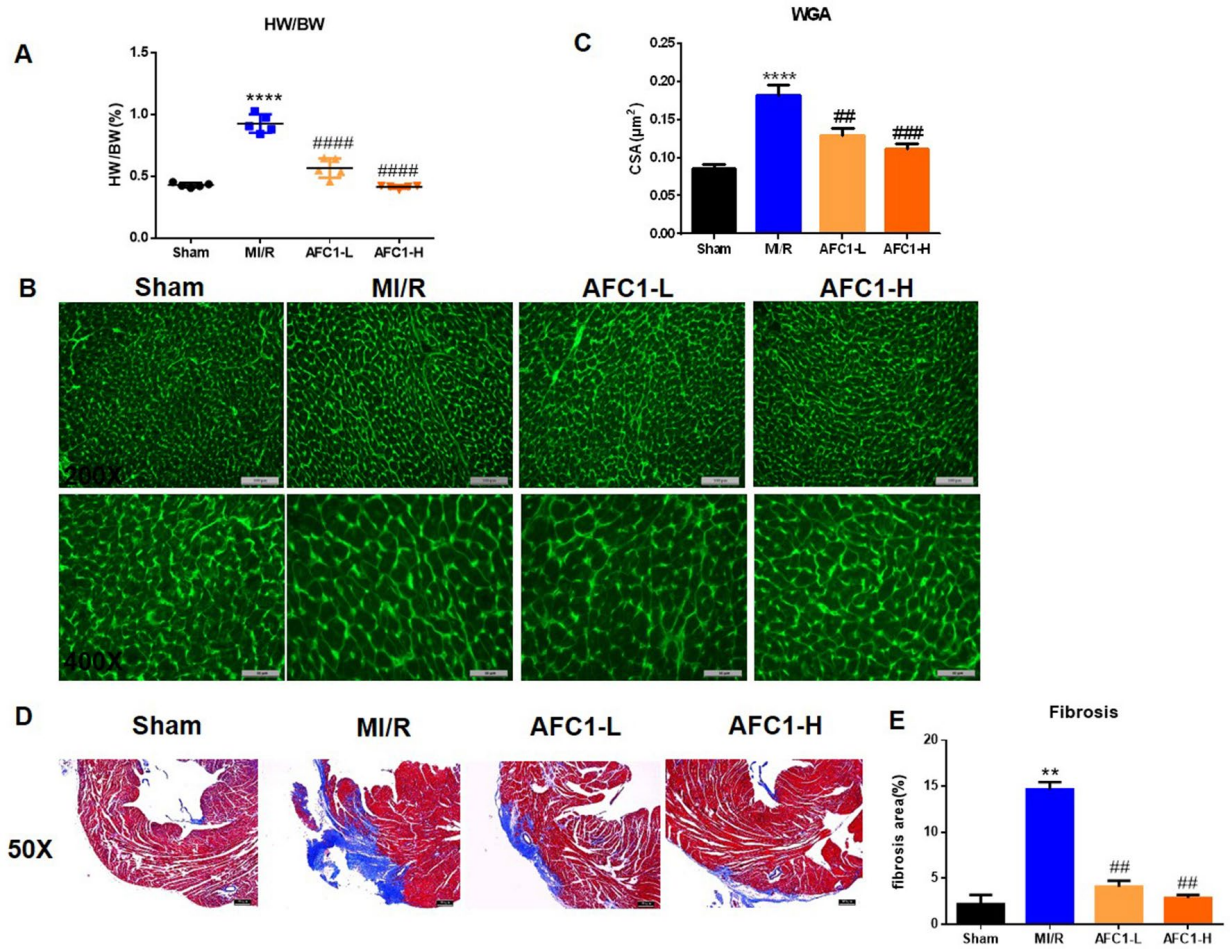
AFC1 Compound attenuated MI/R-induced cardiac remodeling. Heart sections were harvested as mentioned above (n = 5). **(A)** Heart to body weight ratio (HW/BW%) was measured on day 14. **(B)** Representative photomicrographs illustrating ventricular myocyte cross-sections stained WGA. **(C)** Cross-sectional area quantification was shown in statistic graph. **(D)** Hearts were sliced and stained with Masson’s trichrome to assess fibrosis. **(E)** Percent of fibrosis area in each group was shown in statistics. Each experiment was repeated for at least three times and results indicated mean ± SEM of one independent experiment. **p < 0.01, ****p < 0.0001 versus sham; ^##^p < 0.01, ^###^p < 0.001, ^####^p < 0.0001 versus MI/R.

Both dosages of AFC1 reduced the heart to body weight ratio (HW/BW) of mice effectively in comparison to MI/R group on day 14 (p < 0.0001) (**Figure 2A**). WGA staining data revealed significant myocyte hypertrophy in MI/R group compared to the sham (p < 0.0001) and treatment with AFC1 greatly inhibited cardiac hypertrophy (p < 0.01 and p < 0.001) (**Figures 2B, C**). Furthermore, AFC1 treated mice showed alleviated cardiac fibrosis compared to the mice without treatment on day 14 following surgery (**Figures 2D, E**).

### Antioxidant and Anti-Inflammatory Effects of AFC1 Compound on Infarcted Myocardium Following MI/R *In Vivo*

**Figure 3:**
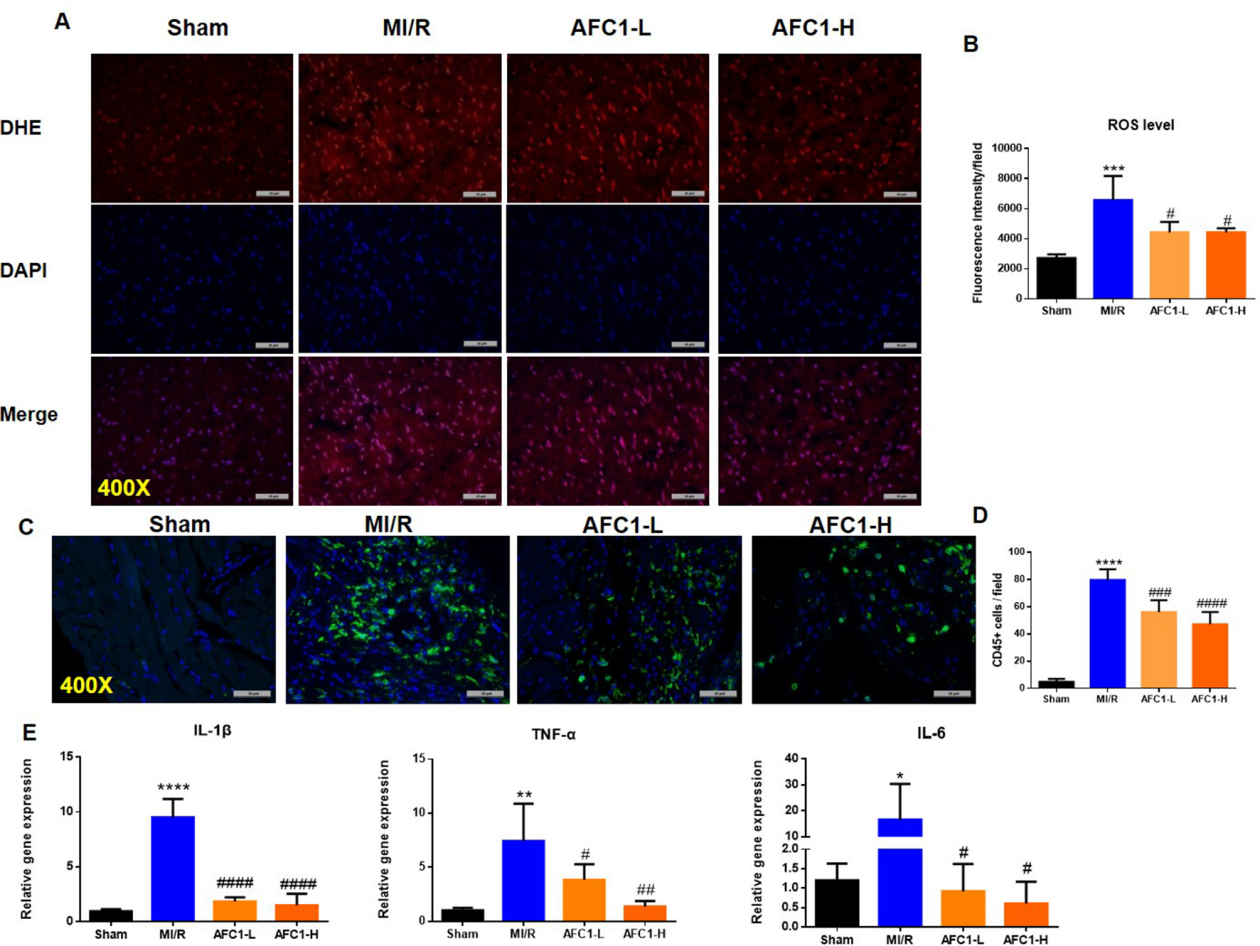
Antioxidant and anti-inflammatory Effects of AFC1 compound on infarcted myocardium following MI/R *in vivo*. Heart sections were harvested and frozen as mentioned above (n = 5). **(A)** Reactive oxygen species production was assessed by dihydroethidium (DHE) conversion to red ﬂuorescent ethidium. **(B)** Fluorescence intensity was evaluated to determine ROS level. **(C)** Immunofluorescence staining of CD45 in transverse section of heart (400×). **(D)** CD45+ cells were quantified in the field. **(E)** Hearts were homogenized and q-PCR was performed to quantify IL-1β, TNF-α, and IL-6 mRNA levels. Results indicated mean ± SEM of one representative experiment and the experiment was repeated three times. *p < 0.05, **p < 0.01, ***p < 0.001, ****p < 0.0001 versus sham; ^#^p < 0.05, ^##^p < 0.01, ^###^p < 0.001, ^####^p < 0.0001 versus MI/R.

It is well established that oxidative stress and inflammation response contribute to cardiac remodeling and dysfunction following MI/R. We then detected the production of ROS in hearts of different groups. MI/R significantly increased the ROS level, while AFC1 treatment greatly decreased ROS accumulation in the infarcted area (p < 0.05) (**Figures 3A, B**). **Figures 3C and D** showed that high dosage of AFC1 dramatically decreased the CD45+ cell infiltrations in heart post MI/R (p < 0.0001). Moreover, both high and low dosages of AFC1 compound significantly down-regulated mRNA levels of inflammatory cytokines including IL-1β, IL-6, and TNF-α in MI/R hearts (**Figure 3E**). These results suggested that AFC1 can attenuate MI/R-induced inflammatory responses in the heart.

### AFC1 Inhibited the Expression of PDGFR in Murine Heart Following MI/R

**Figure 4:**
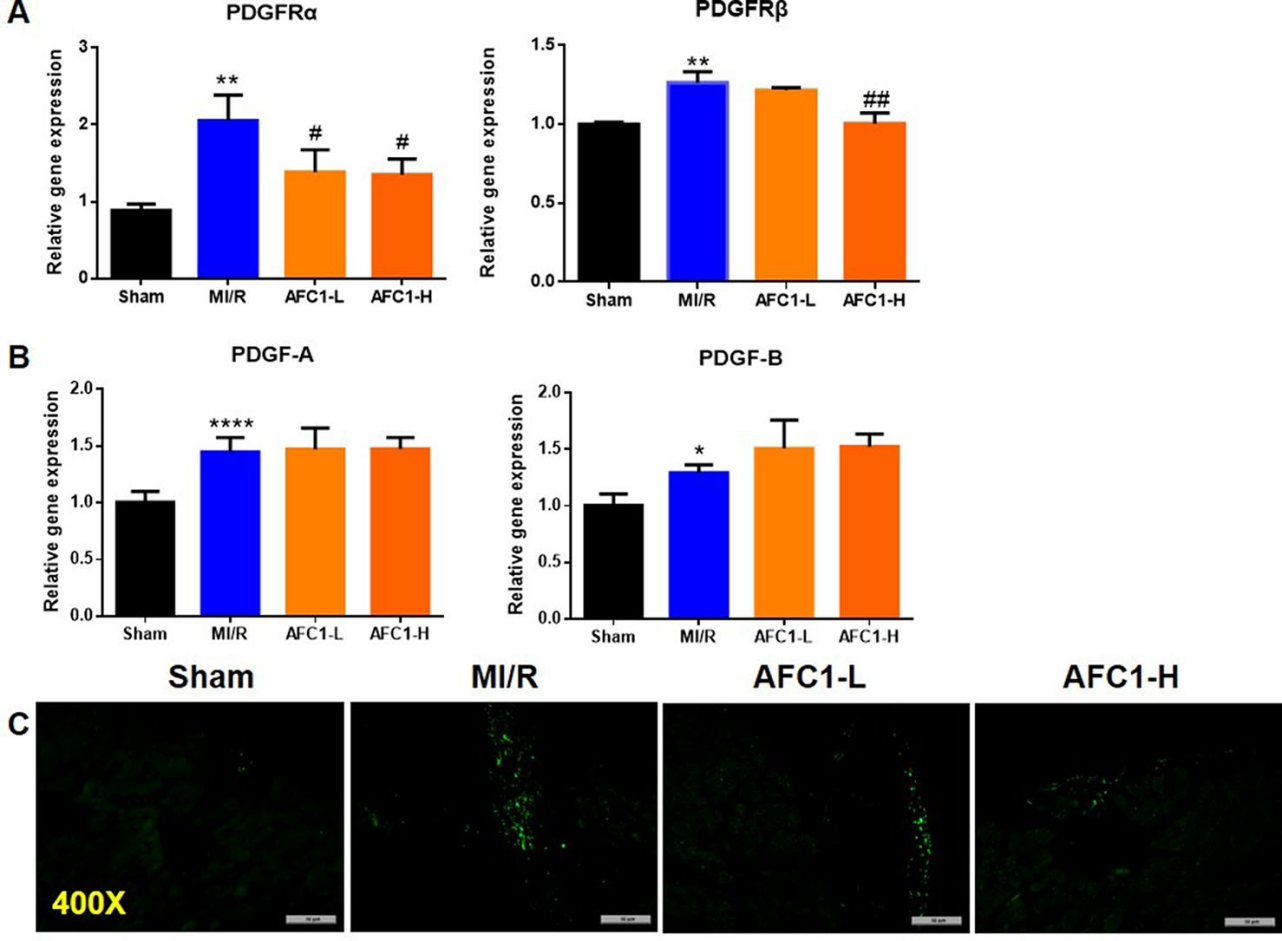
AFC1 inhibited the expression of PDGFR in heart following MI/R (n = 5). Total RNA was extracted from hearts in each group and q-PCR was performed to quantify PDGFRα, PDGFRβ **(A)**, PDGF-A, and PDGF-B **(B)** mRNA levels. **(C)** Immunofluorescence staining of PDGFRα in transverse section of heart (400×). Each experiment was repeated for at least three times and results indicated mean ± SEM of one representative experiment. *p < 0.05, **p < 0.01, ****p < 0.0001 versus sham; ^#^p < 0.05, ^##^p < 0.01 versus MI/R.

Previous study revealed that PDGFs are involved in myocardial remodeling following infarction (Zhao et al., 2011). To verify whether MI/R could lead to an up-regulation of PDGF-related signaling, we measured the expression of PDGF-A, PDGF-B, and PDGFR isoforms in infarcted myocardium. As shown by **Figure 4A**, 30 min of ischemia followed by 2 weeks reperfusion markedly elevated mRNA levels of PDGFRα, -β, PDGF-A, and PDGF-B (**Figures 4A, B**). Next, we treated the MI/R mice with AFC1 and data showed high dose of AFC1 down-regulated both PDGFRα and -β mRNA levels in MI/R hearts. Low dose of AFC1 also decreased the expression of PDGFRα (p < 0.05). However, AFC1 did not affect PDGF-A or PDGF-B expression in hearts subjected to MI/R. Then we determined the PDGFRα protein level by immunofluorescence. As shown in **Figure 4C**, PDGFRα protein expression in the heart increased after MI/R, while it decreased after treatment with AFC1. (Nizamutdinova et al., 2012).

### Effect of PDGF Stimulation on PDGFR Expression in NCM and NCF *In Vitro*

**Figure 5:**
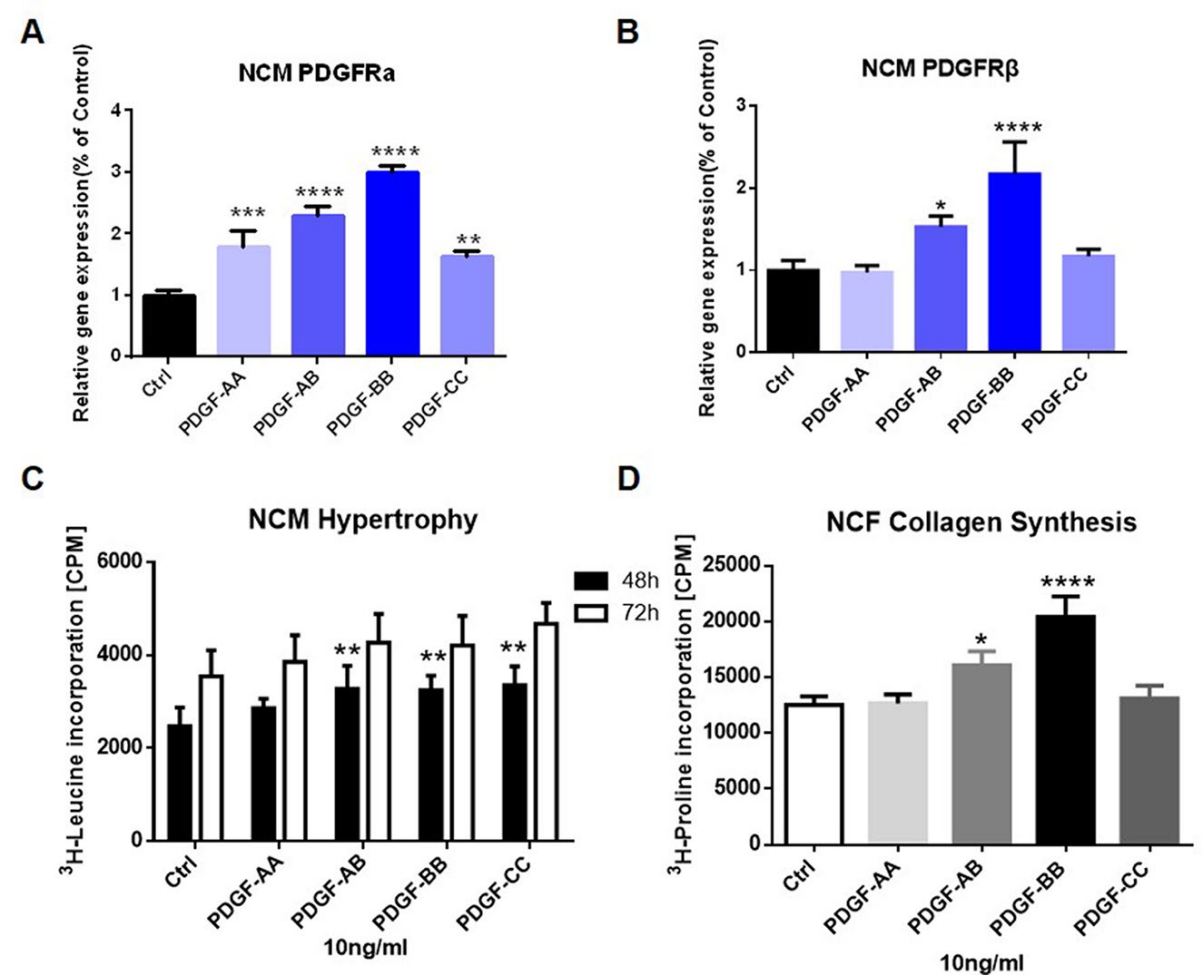
Effect of PDGF stimulation on PDGFR expression in NCM and NCF *in vitro*. PDGFRα **(A)** and PDGFRβ **(B)** mRNA expression in NCM were assessed by q-PCR. NCM hypertrophy **(C)** was measured by ^3^H-leucine incorporation and NCF collagen synthesis **(D)** measured by ^3^H-proline incorporation on different PDGF isoform stimulation. Each experiment was repeated for four times and results indicated mean ± SEM of one representative experiment. *p < 0.05, **p < 0.01, ***p < 0.001, ****p < 0.0001 versus control.

To further elucidate the role of PDGF signaling on heart remodeling, we treated NCM and NCF with different isoforms of PDGF for 48 or 72 h *in vitro*. As shown by **Figure 5A**, addition of PDGF-AA, PDGF-AB, PDGF-BB, or PDGF-CC all increased the mRNA levels of PDGFRα in NCM compared to the control media. Besides, PDGF-AB or PDGF-BB treatment up-regulated the PDGFRβ mRNA expression in NCM (**Figure 5B**). **Figure 5C** showed that NCM stimulated with PDGF-AB, PDGF-BB, or PDGF-CC for 48 h showed significant elevation of protein content in comparison to control cells. Moreover, administration of PDGF-AB or PDGF-BB triggered collagen synthesis in NCF (**Figure 5D**).

### AFC1 Reversed the Elevation of PDGFR Induced by PDGF-AB in Both NCM and NCF *In Vitro*

**Figure 6:**
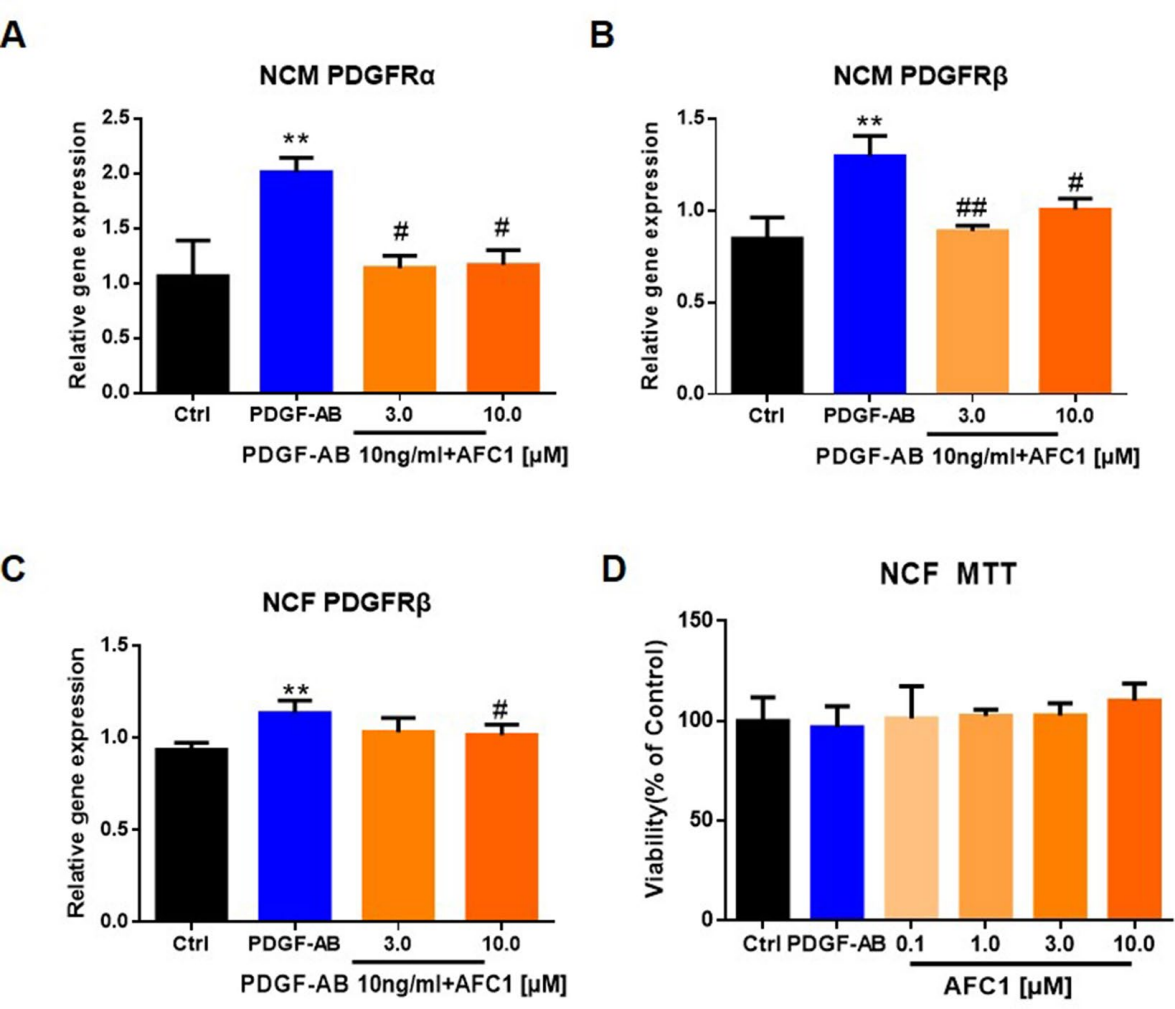
AFC1 reversed the elevation of PDGFR induced by PDGF-AB in both NCM and NCF *in vitro*. PDGFR expression in NCM **(A**, **B)** and NCF **(C)** were quantified by q-PCR. **(D)** Viability of NCF stimulated with PDGF-AB or AFC1 compound (0.1, 1.0, 3.0, 10.0 µM) was evaluated by MTT assay. This experiment was repeated four times and results indicated mean ± SEM of one independent experiment and representative pictures. **p < 0.01 versus control; ^#^p < 0.05, ^##^p < 0.01 versus PDGF-AB.

Next, we treated NCM and NCF with PDGF-AB and AFC1 to determine whether AFC1 compound exerts its protective effect *via* regulating PDGF signaling *in vitro*. As shown by **Figures 6A and B**, addition of AFC1 greatly decreased both PDGFRα and PDGFRβ mRNA expression in NCM stimulated with PDGF-AB. Moreover, high dose of AFC1 reversed the up-regulation of PDGFRβ mRNA levels induced by PDGF-AB in NCF (**Figure 6C**). MTT data (**Figure 6D**) showed that AFC1 did not affect the viabilities of NCF, further confirming that AFC1 inhibits the levels of PDGFRα and PDGFRβ stimulated by PDGF-AB in viable NCF cells.

### AFC1 Suppressed NCM Hypertrophy and NCF Collagen Synthesis Induced by PDGF-AB

**Figure 7:**
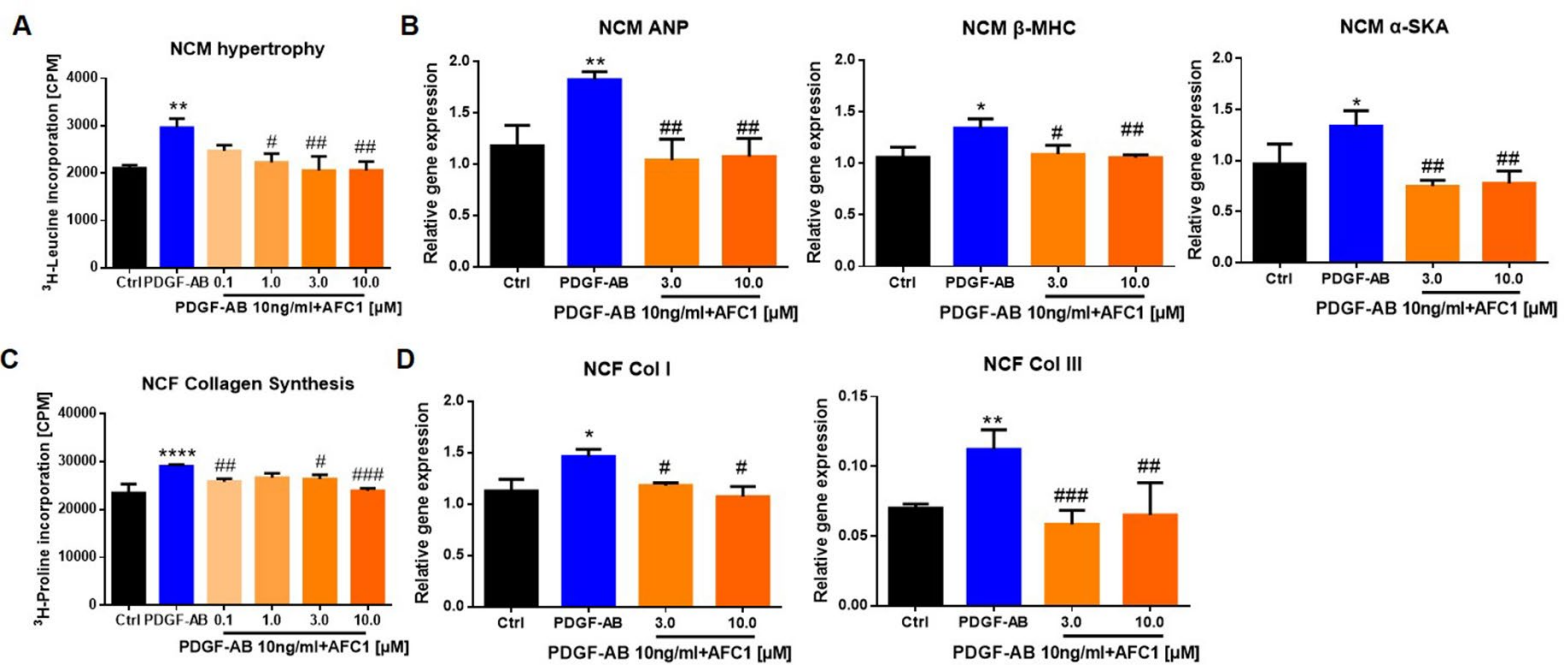
AFC1 suppressed PDGF-AB induced NCM hypertrophy and NCF collagen synthesis. NCM hypertrophy **(A)** was measured by ^3^H-leucine incorporation and NCF collagen synthesis **(C)** measured by ^3^H-proline incorporation on different concentration of AFC1 after PDGF-AB (10 ng/ml) stimulation. mRNA levels of ANP, β-MHC, α-SKA in NCM **(B)**, and Col I, Col III in NCF **(D)** were measured by q-PCR. This experiment was repeated four times and results indicated mean ± SEM of one independent experiment. *p < 0.05, **p < 0.01, ****p < 0.0001 versus control; ^#^p < 0.05, ^##^p < 0.01, ^###^p < 0.001 versus PDGF-AB.

To determine whether AFC1 compound can inhibit PDGF-AB induced NCM hypertrophy and NCF collagen synthesis, we examined the protein content *via* liquid scintillation detector to evaluate cardiac remodeling *in vitro*. **Figure 7A** showed that addition of AFC1 (1.0–10 µM) significantly decreased NCM hypertrophy induced by PDGF-AB in a dose-dependent manner. Furthermore, AFC1 (3.0 and 10 µM) reversed the elevations of ANP, β-MHC, and α-SKA mRNA levels stimulated by PDGF-AB in NCM (**Figure 7B**). AFC1 also inhibited the collagen synthesis (**Figure 7C**) as well as the mRNA expression of Col I and Col III in NCF stimulated by PDGF-AB (**Figure 7D**).

### AFC Modulated Signaling Pathways Involved in Cardiac Function and MI/R

**Figure 8:**
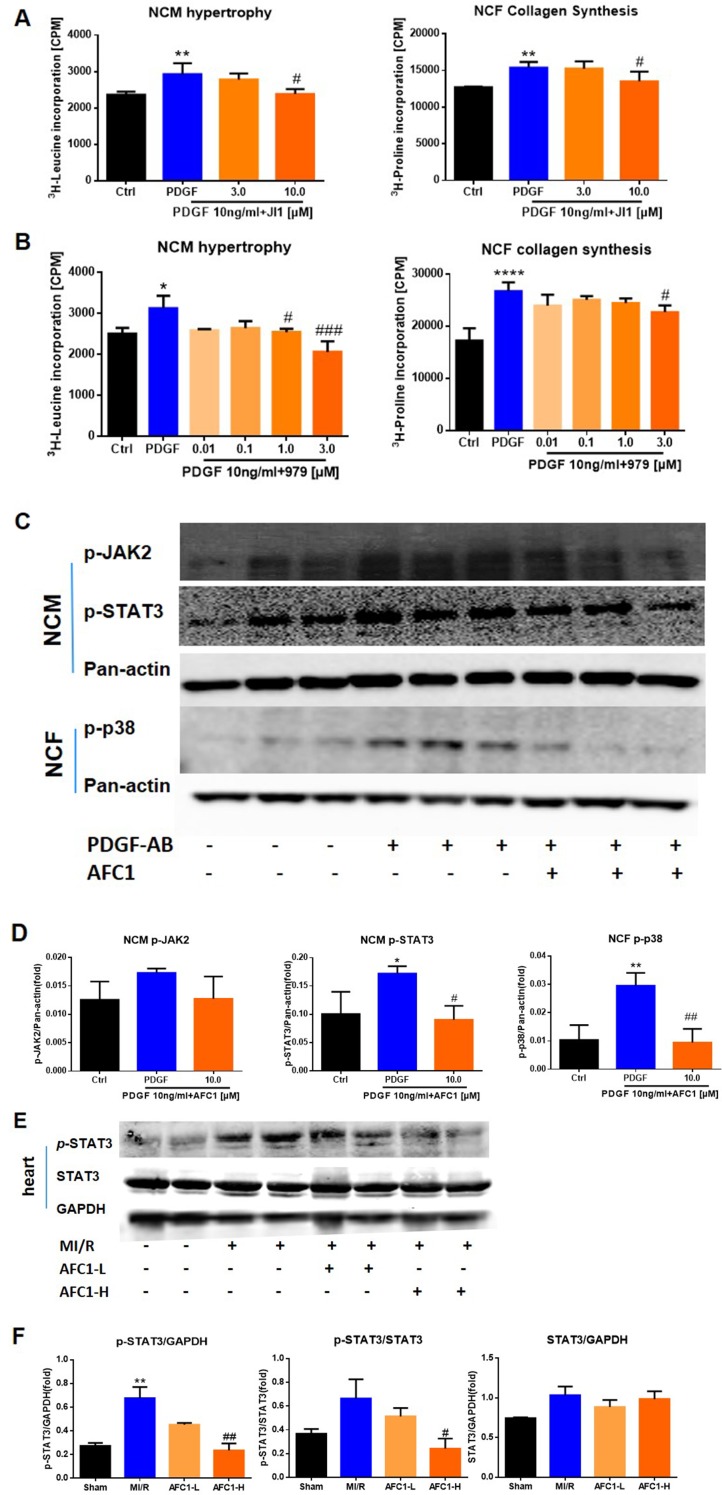
Potential mechanism of the protective function of AFC1 treatment *in vitro* and *in vivo* n = 5. After being treated with JI1 **(A)** or 979 **(B)** and then stimulated with PDGF-AB, NCM hypertrophy was measured by ^3^H-leucine incorporation and NCF collagen synthesis measured by ^3^H-proline incorporation. **(C**, **D)** Protein levels of p-JAK2, p-STAT3 in NCM and p-p38 in NCF were determined *via* Western blot analysis. **(E)** Infarct area of heart in each group was lysed and p-STAT3, STAT3 protein levels were determined *via* Western blot. **(F)** p-STAT3/STAT3, p-STAT3/GAPDH, and STAT3/GAPDH were analyzed with image J software. Two or three samples were randomly selected from each group for Western blot experiment. Each experiment was repeated for at least three times and results indicated mean ± SEM of one independent experiment and the representative pictures. *p < 0.05, **p < 0.01, ****p < 0.0001 versus control or sham; ^#^p < 0.05, ^##^p < 0.01, ^###^p < 0.001 versus PDGF-AB or MI/R.

Previous reports revealed that STAT signaling pathway played a vital role in myocardial remodeling (Aboulhoda, 2017, Wincewicz and Sulkowski, 2017), and P38MAPK pathway is involved in cellular inflammatory response and apoptosis under the condition of ischemia and hypoxia (Owona et al., 2013). We cultured NCM and NCF with PDGF-AB with or without JAK inhibitor JI1 (JAKs inhibitor I-Calbiochem, Darmstadt, Germany) and p38 inhibitor 979 (Cai et al., 2018). As shown by **Figures 8A and B**, both 10 μM JI1 and 3 μM 979 inhibited NCM hypertrophy and NCF collagen synthesis induced by PDGF-AB. Western blots analysis of protein expression of p-JAK2, p-STAT3, and p-p38 in cells revealed that PDGF-AB treatment greatly activated the phosphorylation of STAT3 in NCM and p-38 in NCF. Addition of AFC1 dramatically decreased the levels of p-STAT3 in NCM and p-p38 in NCF induced by PDGF-AB (**Figures 8C, D**). No significant difference was observed in the expression of p-JAK2 in these groups.

For *in vivo* study, MI/R increased the ratio of p-STAT3/GAPDH without affecting p-STAT3/STAT3 and STAT3/GAPDH in infarcted myocardium. Treatment with high dose of AFC1 markedly reversed the elevation of both p-STAT3/GAPDH and p-STAT3/STAT3 without affecting the ratio of STAT3/GAPDH (**Figures 8E, F**).

## Discussion

Early and effective intervention strategies have greatly decreased the mortality of IHD; however, reperfusion could cause myocardium damage, even exacerbate the cardiac function and structure (Wang et al., 2015b). Innovative pharmacotherapies to improve the outcomes especially left ventricular remodeling in patients who suffered from MI are still an urgent need (Della Rocca et al., 2012; Chew et al., 2018). Recently, TCMs have received much attention due to their functions in reduction of myocardial injury (Mo et al., 2015; Yang et al., 2016). Many studies demonstrated the cardioprotective effect of TSA and sodium TSA sulfonate in myocardial ischemia/reperfusion injury animal models (Zhang et al., 2010; Zhang et al., 2013; Wei et al., 2014; Li Q et al., 2016; Pan et al., 2017). Results from the recent clinical trial indicated that sodium TSA sulfonate in combination with current therapies may significantly reduce adverse LV remodeling and potentially improve clinical outcomes, providing important evidence on the efficacy of sodium TSA sulfonate treatment in patients (Mao et al., 2015a; Mao et al., 2015b). Another trail named “Sodium Tanshinone IIA Sulfonate in Left Ventricular Remodeling Secondary to Acute Myocardial Infarction” is going on in Guangzhou, China (ClinicalTrials.gov Identifier: NCT02524964)(https://www.clinicaltrials.gov).

We have been searching new TCM-based cardioprotective agents and identified series AFC compounds with TSA mimic effects. In this study, we reported for the first time the cardioprotective actions of AFC1 in murine MI/R models. Ventricular remodeling including cardiac hypertrophy and fibrosis is the leading cause contributing to cardiac dysfunction after MI/R (Konishi et al., 2013). The key findings of the present study are that administration of AFC1 effectively reduced the cardiac myocyte hypertrophy and fibrosis on day 30 post MI/R, accompanied by significant improvement of cardiac function, and also inhibited MI/R-induced cardiac remodeling *in vivo*. The effect of AFC1 may be mediated by its inhibition of production of ROS and inflammation mediators and regulation of key signaling pathways, including PDGF, STAT3, and p38 signaling pathways.

MI/R is a complex multifactorial pathophysiological process that oxidative stress and inflammatory response are key contributors to the following cardiac remodeling. The damage of oxygen free radical on vessel and myocardium after perfusion could result in myocardial injury and finally accelerate the development of MI/R (Duan et al., 2015). MI/R activated sterile inflammatory response characterized by the recruitment and activation of immune cells (Yan et al., 2013). Numerous studies have demonstrated that inhibition of neutrophil recruitment mediated by macrophage reduced tissue damage and infarct size in ischemic myocardium (Romson et al., 1983; de Lorgeril et al., 1989; Hatori et al., 1991; Li et al., 2016; Wang et al., 2016). Excessive neutrophil infiltration in the infarct site is detrimental to cardiomyocyte survival for secreting ROS, which further aggravates structural damage of tissue (Hansen, 1995). Our data showed that AFC1 compound inhibited the production of ROS, the infiltration of inflammatory cells, as well as the content of inflammatory cytokines, such as IL-1β, TNF-α, and IL-6 in infarcted myocardium, which implied that AFC1 compound may play an important role in improving ventricular remodeling after MI/R *via* suppressing oxidative stress and inflammatory responses.

Previously, evidences indicated the crucial role of PDGF on stimulating fibrosis in many pathological conditions (Choi et al., 2005; Fan et al., 2013). Hypoxia could cause murine pulmonary vascular medial hypertrophy *via* increasing PDGF concentration (Zhang et al., 2012). PDGF-AB released by myofibroblast could cause myocyte structural and electromechanical remodeling in ovine persistent atrial fibrillation (PAF) through reducing calcium transients (Musa et al., 2013). The expression of PDGF is closely related to inflammation and fibrosis in infarcted myocardium (Zhao et al., 2011). In addition, PDGF can induce ROS production in MEFs (Choi et al., 2005). Therefore, PDGF may induce cardiac remodeling following MI/R *via* promoting oxidative stress and inflammatory response. The present result showed much higher expressions of PDGF-A, PDGF-B, PDGFRα, and PDGFRβ in murine MI/R myocardium in MI/R heart than that in the sham group. Previous report from the MI rat models (Liu et al., 2014) revealed similar findings. Our results further indicated that AFC1 compound can effectively decrease the levels of PDGFR without affecting the expression of PDGF-A and PDGF-B in the heart post MI/R. Besides, AFC1 treatment greatly reduced PDGF-AB-stimulated PDGFR mRNA expression in NCM and NCF, as well as inhibited PDGF-AB-stimulated NCM hypertrophy and NCF collagen synthesis *in vitro*. These data suggested that cardio-protective function of AFC1 may attribute to the inhibition of PDGFR signaling *in vivo* and *in vitro*.

It is proved that Janus kinase/signal transduction and activators of transcription (JAK/STAT) pathway can be activated by ischemic stress stimuli and cardiac hypertrophy agonist PDGF (Goodman et al., 2011; Wu et al., 2012). Tyrosine kinase could phosphorylate receptor tyrosine residues expressed on cardiomyocytes and activate the STAT phosphorylation. The activated STAT then transferred to nucleus and bonded to the target gene to regulate the expression of transcription factors or genes associated with hypertrophy and fibrosis, such as p21waf1 and c-fibrinogen (Wagner and Siddiqui, 2012). Inhibition of JAK/STAT pathway could reduce the myocardial infarct size and cardiomyocyte apoptosis induced by MI/R in rat models (Mascareno et al., 2001). Besides, PDGF-AB stimulated proliferation of human airway smooth muscle cells, which contribute to airway remodeling through the JAK/STAT pathway (Simon et al., 2002). Studies revealed that the PDGF/PDGFR pathway is involved in the regulation of cardiac function and the development of ventricular remodeling in MI/R *via* JAK/STAT downstream pathway (Wang et al., 2000; Booz et al., 2002). On the other hand, as an important intracellular signaling enzyme, P38MAPK is activated by myocardial ischemia and hypoxia to induce cellular apoptosis, and results in impaired cardiac function and amplifies the inflammatory cascade in the heart following MI/R. Our *in vitro* data showed that stimulation of PDGF-AB increased STAT3 phosphorylation in NCM and p38 phosphorylation in NCF, and addition of AFC1 compound significantly decreased both proteins’ phosphorylation, as well as suppressed NCM hypertrophy and NCF collagen synthesis. Moreover, in murine MI/R models, the expression of p-STAT3 up-regulated in murine infarcted myocardium and this elevation can be dramatically decreased by AFC1 treatment, indicating the possible downstream pathway in which AFC1 exerts its role in cardiac myocyte. We therefore proposed that AFC1 compound may attenuate MI/R-induced cardiac remodeling *via* regulating PDGFR signaling and inhibiting the phosphorylation of STAT3. It is also possible that AFC1 may act on other singling pathways, but further study is needed to elucidate this.

Importantly, AFC1 showed neither detrimental impact on morphological and histological changes of murine lung, liver, kidney, and spleen, nor the cytotoxicity in HUVEC viability. It showed less cytotoxicity than TSA in high doses, indicating it may have a better safety profile than TSA. Since TSA has been widely used clinically with excellent safety profile, AFC1 may also be a potential clinical agent for treating MI/R. But further study is needed on its pharmacokinetic profile and more detailed evaluation of its toxicity *in vivo*. In addition, further investigations are needed to explore the role of AFC1 in other cardiovascular diseases and further in clinical trials.

## Conclusions

AFC1 compound had comparable effect with TSA in improving cardiac function after MI/R. Administration of AFC1 suppressed STAT signaling and attenuated MI/R-induced cardiac remodeling in murine MI/R models. AFC1 suppressed PDGF-AB induced NCM hypertrophy *via* STAT3 pathway and NCF collagen synthesis through p38 signaling. Therefore, AFC1 may be a novel therapeutic option with anti-hypertrophic and anti-fibrotic effect against MI/R-induced cardiac remodeling in patients who suffered from MI/R.

## Data Availability Statement

The raw data supporting the conclusions of this manuscript will be made available by the authors, without undue reservation, to any qualified researcher.

## Ethics Statement

This study was carried out in accordance with the recommendations of the Institutional Animal Care and Use Committee of Tongji University. The protocol was approved by the Institutional Animal Care and Use Committee of Tongji University.

## Author Contributions

XZ, BW, and HF contributed to the conception and design of the study. JieL, XZ, QM, KH, JingL, JT, RZ, GC, YZ, LW, and LH contributed to acquisition, analysis, and interpretation of the data. JieL, XZ, CL, ZL, and BW wrote and revised the MS.

## Funding

The study was supported by the National Key Research and Development Program of China (2017YFA0105600), the National Natural Science Foundation of China (81370434; 81670458; 81470393), the Shanghai Municipal Health and Family Planning Commission (ZY3-LCPT-2-1003-2014ZYJB0502), Key Discipline Project of Pudong Health Bureau of Shanghai (PWZxk2017-01), and the Science and Technology Commission of Shanghai Municipality (17431906600).

## Conflict of Interest

The authors declare that the research was conducted in the absence of any commercial or financial relationships that could be construed as a potential conflict of interest.
